# Secretogranin III Selectively Promotes Vascular Leakage in the Deep Vascular Plexus of Diabetic Retinopathy

**DOI:** 10.3390/ijms241310531

**Published:** 2023-06-23

**Authors:** Liyang Ji, Prabuddha Waduge, Yan Wu, Chengchi Huang, Avinash Kaur, Paola Oliveira, Hong Tian, Jinsong Zhang, J. Timothy Stout, Christina Y. Weng, Keith A. Webster, Wei Li

**Affiliations:** 1 Cullen Eye Institute, Department of Ophthalmology, Baylor College of Medicine, Houston, TX 77030, USA; 2 Bascom Palmer Eye Institute, University of Miami School of Medicine, Miami, FL 33136, USA; txwuyan@163.com; 3 Everglades Biopharma, LLC, Houston, TX 77098, USA; 4 Department of Ophthalmology, The Fourth Affiliated Hospital of China Medical University, Shenyang 110005, China; cmuzhangjs@163.com

**Keywords:** diabetic retinopathy, endothelial heterogeneity, Scg3, VEGF, disease-targeted anti-angiogenic therapy, synergistic combination anti-angiogenic therapy

## Abstract

Diabetic retinopathy (DR), a leading cause of vision loss in working-age adults, induces mosaic patterns of vasculopathy that may be associated with spatial heterogeneity of intraretinal endothelial cells. We recently reported that secretogranin III (Scg3), a neuron-derived angiogenic and vascular leakage factor, selectively binds retinal vessels of diabetic but not healthy mice. Here, we investigated endothelial heterogeneity of three retinal vascular plexuses in DR pathogenesis and the therapeutic implications. Our unique in vivo ligand binding assay detected a 22.7-fold increase in Scg3 binding to retinal vessels of diabetic mice relative to healthy mice. Functional immunohistochemistry revealed that Scg3 predominantly binds to the DR-stressed CD31^−^ deep retinal vascular plexus but not to the relatively healthy CD31^+^ superficial and intermediate plexuses within the same diabetic retina. In contrast, VEGF bound to healthy and diabetic retinal vessels indiscriminately with low activity. FITC-dextran assays indicated that selectively increased retinal vascular leakage coincides with Scg3 binding in diabetic mice that was independent of VEGF, whereas VEGF-induced leakage did not distinguish between diabetic and healthy mice. Dose–response curves showed that the anti-Scg3 humanized antibody (hAb) and anti-VEGF aflibercept alleviated DR leakage with equivalent efficacies, and that the combination acted synergistically. These findings suggest: (i) the deep plexus is highly sensitive to DR; (ii) Scg3 binding to the DR deep plexus coincides with the loss of CD31 and compromised endothelial junctions; (iii) anti-Scg3 hAb alleviates vascular leakage by selectively targeting the DR-stressed deep plexus within the same diabetic retina; (iv) combined anti-Scg3 and anti-VEGF treatments synergistically ameliorate DR through distinct mechanisms.

## 1. Introduction

Heterogeneity of the vascular endothelium relating to different organs, locations (artery, arteriole, capillary, venule and vein), structures (regular, fenestrated and sinusoidal), and functions (endocytosis, adhesion and permeability) is well documented [1]. Single-cell RNA-seq (scRNA-seq) confirms such heterogeneity at the level of gene expression [2]. Endothelial cell (EC) heterogeneity and related dysfunction are features of multiple diseases, including diabetes, coronary artery disease and calcific aortic valve sclerosis, and constitute important pharmacological targets [3,4].

Diabetic vascular complications systemically manifest in multiple organs, and diabetic retinopathy (DR), a leading cause of vision loss in working-age adults, afflicted more than 103.1 million people worldwide in 2020 [5]. DR is characterized by dysfunctional neurovascular units with mosaic vasculopathy in the retina, including altered blood flow, vascular leakage, retinal thickening and acellular capillaries, and may progress to microaneurysms, retinal hemorrhages, diabetic macular edema (DME) and proliferative DR (PDR) [6,7]. Vascular endothelial growth factor (VEGF) inhibitors, such as ranibizumab and aflibercept, are approved for DR therapy but with a limited efficacy to improve visual acuity [6,8,9].

The human retinal vasculature comprises three layers of microcapillary networks: the superficial, intermediate and deep plexuses that vascularize the inner half of the retina [10]. Photoreceptors in the outer half of the avascular retina are the most metabolically active cells in the retina and rely heavily on diffusion of nutrients, especially oxygen and glucose, from the retinal plexuses and choriocapillaris [6,11,12]. The deep plexus located adjacent to the photoreceptors is implicated as an injury-susceptible vasculature wherein abnormalities correlate most closely with DR pathogenesis in patients [13]. However, delineation of the molecular pathways that drive DR progression within the different plexuses is relatively unexplored.

Using a newly developed technology called ligandomics [14], we recently discovered secretogranin III (Scg3) as a diabetes-selective angiogenic and vascular leakage factor that preferentially binds to diabetic over healthy retinal vasculatures [15]. Functional analyses independently confirmed that Scg3 induces angiogenesis and retinal vascular leakage selectively in diabetic mice, whereas VEGF promotes angiogenesis and leakage indiscriminately [15,16]. Scg3-neutralizing monoclonal antibodies (mAbs) alleviate DR leakage with high efficacy in diabetic mice [15]. Anti-Scg3 humanized antibodies (hAbs) also inhibit pathological but not physiological angiogenesis in animal models of retinopathy of prematurity (ROP) [17,18]. While theoretically all retinal vessels are exposed to the same level of hyperglycemia, the biological consequences of such effects may be context-dependent, i.e., determined by spatial and functional endothelial heterogeneity within the retinal plexuses. Such heterogeneity may provide an avenue for disease-targeted anti-angiogenic or anti-leakage therapy of DR within the same diabetic retina.

The primary objectives of this study were to investigate differential binding of Scg3 to individual intraretinal vasculatures in diabetic versus non-diabetic mice, compare binding patterns with those of VEGF, and relate the binding to endothelial dysfunction and disease-targeted therapy with anti-Scg3 antibodies. Additionally, as a prelude to a possible improvement of treatment efficacy, we report synergistic combination therapy of anti-Scg3 and anti-VEGF through distinct mechanisms of action.

## 2. Results

### 2.1. Constitutive Scg3 Expression in Diabetic and Healthy Retinas

**Figure 1:**
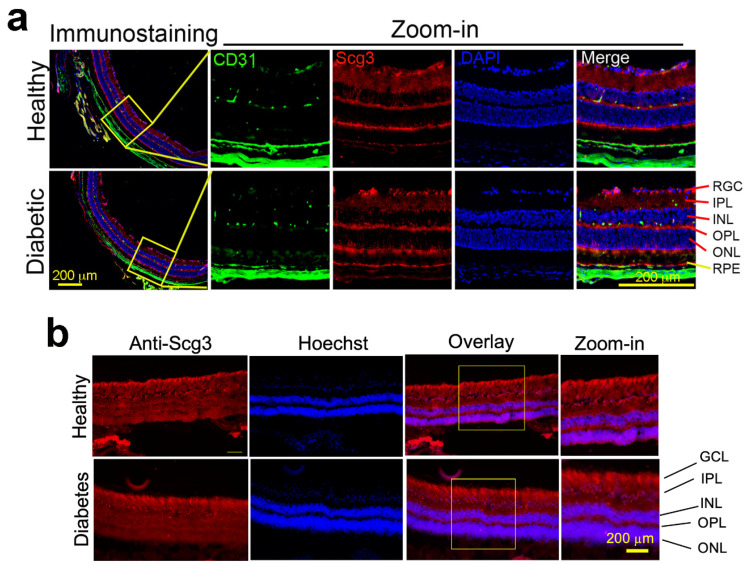
Constitutive expression of Scg3 in diabetic and healthy retinas of mice and humans. (**a**) Diabetic and healthy mouse retinas. Immunohistochemistry to detect Scg3 expression in the retinas of healthy and diabetic mice. Scg3 signals (red) are reduced in nuclear layers. CD31 signals (green) is an endothelial marker. Blue signals are for nuclei. (**b**) Diabetic and healthy human retinas. Scg3 expression is not upregulated in the diabetic mice and human retinas. Scale = 200 µm. RGC, retinal ganglion cells; IPL, inner plexiform layer; INL, inner nuclear layer; OPL, outer plexiform layer; ONL, outer nuclear layer; RPE, retinal pigment epithelium. Yellow boxes are the areas for zoom-in images.

We previously reported that Scg3 levels changed only minimally in the retinas of diabetic mice, as detected by Western blots [15]. Here, we compared Scg3 expression in the diabetic and healthy retinas by immunohistochemistry and confirmed similar levels of expression in both conditions in mice and humans (Figure 1). Scg3 is predominantly expressed in the retinal ganglion cell (RGC) layer, inner plexiform layer (IPL), outer plexiform layer (OPL), photoreceptor inner segments and retinal pigment epithelium (RPE), but minimally in the inner and outer nuclear layers and photoreceptor outer segments. The specificity of immunostaining signals was confirmed using wild-type and Scg3-knockout retinas (Supplementary Figure S1).

### 2.2. Scg3 Selectively Induces Vascular Leakage in Diabetic but Not Healthy Retinas

**Figure 2:**
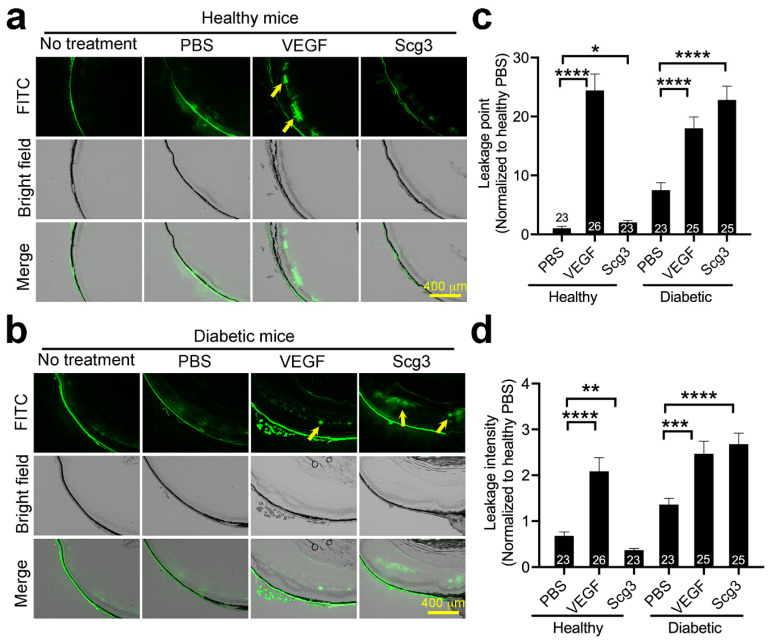
VEGF and Scg3 differentially stimulate retinal vascular leakage in healthy and diabetic mice. (**a**,**b**) Representative images of FITC-dextran (70 kDa, green) in vivo leakage in healthy (**a**) and diabetic (**b**) mice receiving intravitreal injection of hVEGF (100 ng/1 µL/eye), hScg3 (250 ng/1 µL/eye) or PBS. Arrows indicate leakage spots. (**c**) Quantification of leakage spots in (**a**,**b**). (**d**) Quantification of leakage intensity in (**a**,**b**). Sample sizes (number of viewing fields/group) is indicated in the graphs (5 mice/group). ±SEM, * *p* < 0.05, ** *p* < 0.01, *** *p* < 0.001, **** *p* < 0.0001; one-way ANOVA test.

To compare relative levels of VEGF- versus Scg3-induced retinal vascular leakage, healthy and diabetic mice pretreated with ivt VEGF or Scg3 were injected i.v. with FITC-dextran, and FITC leakage from the retinal vasculature was quantified. The results indicated that VEGF induced retinal vascular leakage equally in both healthy and diabetic mice, whereas Scg3 stimulated retinal leakage only in diabetic mice (Figure 2). The findings suggest that Scg3, in contrast to VEGF, is a highly diabetes-restricted retinal vascular leakage factor.

### 2.3. Scg3 Selectively Binds to Diabetic Retinas but Not Other Organs

**Figure 3:**
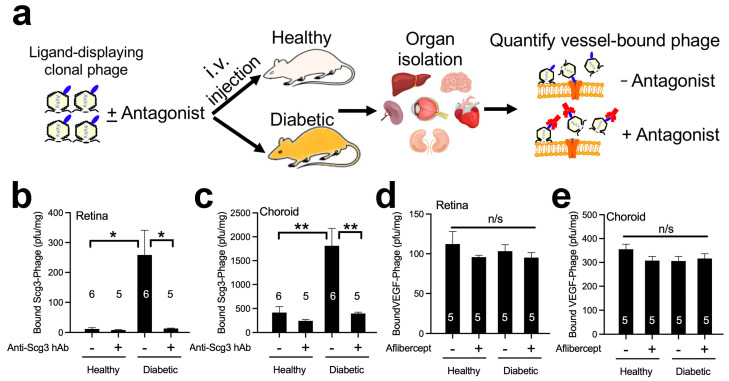
Increase in Scg3 binding to diabetic but not healthy retinal vessels. (**a**) Schematic of in vivo ligand binding assay in live mice to quantify ligand binding to healthy and diabetic organs with or without blockade by ligand-specific antagonist. (**b**,**c**) Scg3-Phage binding to the retina (**b**) or choroid (**c**) in diabetic or healthy mice in the presence or absence of anti-Scg3 hAb. (**d**,**e**) VEGF-Phage binding to the retina (**d**) or choroid (**e**) in diabetic or healthy mice with or without aflibercept blocking. Sample sizes (mice/group) are indicated in the graph in (**b**–**e**). ±SEM; * *p* < 0.05, ** *p* < 0.01; n/s, not significant; one-way ANOVA test.

We proposed in a previous review that pathological angiogenesis and vascular leakage may be driven by upregulation of angiogenic ligands, their receptors or both [19]. To investigate the possibility that Scg3 receptor (Scg3R) availability rather than ligand abundance drives Scg3-mediated vascular leakage, we quantified and compared the binding of Scg3 and VEGF to healthy and diabetic retinas using clonal T7 phages displaying VEGF (VEGF-Phage) or Scg3 (Scg3-Phage). Clonal phages ± pre-blocking reagents were injected i.v. into diabetic and healthy mice, as indicated (Figure 3a). After 20 min of binding and intracardial perfusion to remove unbound phages, retinas and other organs were isolated, homogenized and quantified for vessel-bound phages. We found a 22.7-fold increase in binding of Scg3-Phage to diabetic retinal vessels relative to healthy retinal vessels that were blocked by anti-Scg3 hAb (Figure 3b). A similar increase in Scg3-Phage binding was recorded in the choroid of diabetic mice, but not in other organs, including the kidney, heart, spleen and liver (Figure 3c and Supplementary Figure S2). In contrast, VEGF-Phage binding was not increased in any of the tested diabetic organs, including the retina, choroid, heart, liver or spleen (Figure 3d,e and Supplementary Figure S3). VEGF-specific binding was significantly blocked by aflibercept in healthy hearts (27.2% blocked) and diabetic kidneys (48.1% blocked) and hearts (47.2% blocked) (Supplementary Figure S3). However, aflibercept-sensitive binding activities were significantly less than the equivalent anti-Scg3-blocked binding to diabetic retinas (Figure 3b vs. Figure 3d). These findings are consistent with our previous report that Scg3 is a diabetes-restricted vascular binding and leakage factor [15,16].

### 2.4. Scg3 Preferentially Binds to the Deep Vascular Plexus in Diabetic Retinas

**Figure 4:**
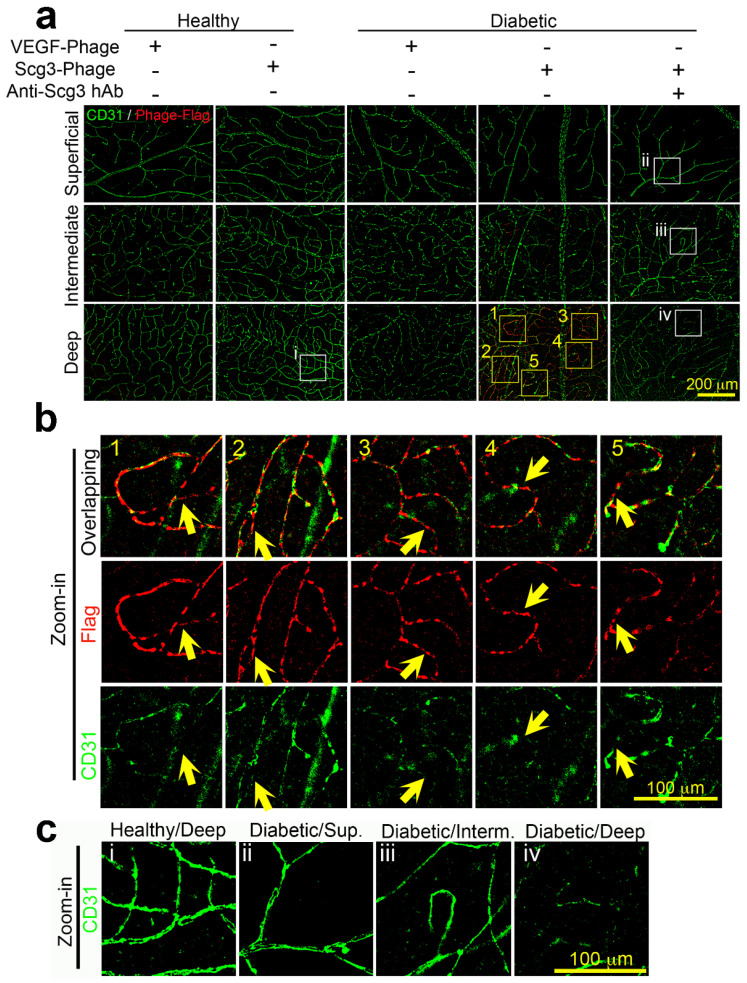
Functional immunohistochemistry (FIHC) of Scg3 binding only to the deep plexus of the retinal vasculature in diabetic but not healthy mice. (**a**) Binding of clonal Scg3- or VEGF-Phage to superficial, intermediate and deep plexuses of healthy and diabetic retinas in the presence or absence of anti-Scg3 hAb. (**b**) Zoom-in Scg3 binding signals and CD31+ endothelial cells. Arrows indicate CD31-negative microcapillaries with Scg3 binding signals. Column 1-5 in (**b**) are zoom-in images for Box 105 in (**a**), as indicated by cognate numbers. (**c**) CD31 is markedly down regulated in the diabetic deep plexus but minimally in other plexuses of healthy and diabetic retina. White Box i-iv are areas for zoom-in images in (**c**), as indicated by cognate numbers. Red signals indicate vessel-bound Scg3- or VEGF-Phage; green signals indicate CD31 on retinal endothelial cells.

To investigate retinal endothelial heterogeneity, we first examined the integrity of the superficial, intermediate and deep plexuses. Immunohistochemistry against CD31 (PECAM-1), a well-known endothelial marker and endothelial junction protein, revealed that all three layers of retinal vascular networks were clearly visible in healthy retinas, as were the superficial and intermediate plexuses in the diabetic retina (Figure 4a,c). However, CD31 expression in the deep plexus of 4-month-diabetic mice was markedly downregulated, suggesting stress and an increased endothelial permeability of the deep plexus (Figure 4a,b). In some areas of the deep plexus, CD31 staining was so faint that continuous microcapillary networks were not readily discernable, suggesting a disruption of endothelial integrity that may contribute to DR leakage (Figure 4).

Because Scg3R has not yet been identified, conventional immunohistochemistry cannot be used to directly measure receptor densities on diabetic versus healthy retinal vasculatures. Therefore, we developed a technique designated FIHC to visualize ligand binding locations on vasculatures by combining in vivo ligand binding with immunohistochemistry. FIHC using anti-FLAG mAb confirmed that FLAG-tagged Scg3-Phage bound predominantly to the deep plexus of diabetic retinas and minimally to the diabetic intermediate plexus. By this method, Scg3 binding signals were not detected in the diabetic superficial plexuses or healthy plexuses. Interestingly, Scg3 binding was markedly increased in microcapillaries that coincided with the most severe loss of CD31 signals, but with only minimal changes in regions of normal apparent CD31 (Figure 4a,b). Superimposition of Scg3 staining on microcapillary areas with discontinuous CD31 signals presented reconstituted images of continuous microcapillary networks (Figure 4a,b). Magnified images indicated contiguous but non-overlapping Scg3 binding (red) and CD31 signals (green), in the deep plexus (Figure 4b), suggesting negative interference of CD31 integrity by Scg3R upregulation.

Interestingly, weak but elevated Scg3 binding signals were visible in the deep plexus of 2- and 3-month-diabetic mice (Supplementary Figure S4). Sporadic but increasing Scg3 binding signals were also detected in the intermediate plexus of 2- to 4-month-diabetic mice (Figure 4a and Supplementary Figure S4). The results suggest that an initially enhanced binding of Scg3 to the deep retinal plexus spreads to the intermediate plexus during the progression of DR.

In contrast, FIHC detected minimal VEGF-Phage binding to any vascular plexuses in healthy or DR retinas (Figure 4a), consistent with the indiscriminate and minimal VEGF-specific binding (Figure 3d). Together, the results are consistent with selective binding of Scg3 but not VEGF to DR vessels.

### 2.5. Neutralizing Activity of anti-Scg3 hAb

We previously generated and characterized Scg3-neutralizing mAb that alleviated DR leakage in diabetic mice with high efficacy [15]. For the present study, and for eventual clinical application, we engineered an anti-Scg3 hAb and independently characterized the neutralizing activity of the new anti-Scg3 reagent in terms of transwell migration, permeability TEER and tube formation using HRMVECs (Supplementary Figures S5–S8). The results confirmed that the neutralizing activities are comparable with those of aflibercept.

### 2.6. Scg3 and Aflibercept Alleviate DR Leakage with Similar High Efficacy

**Figure 5:**
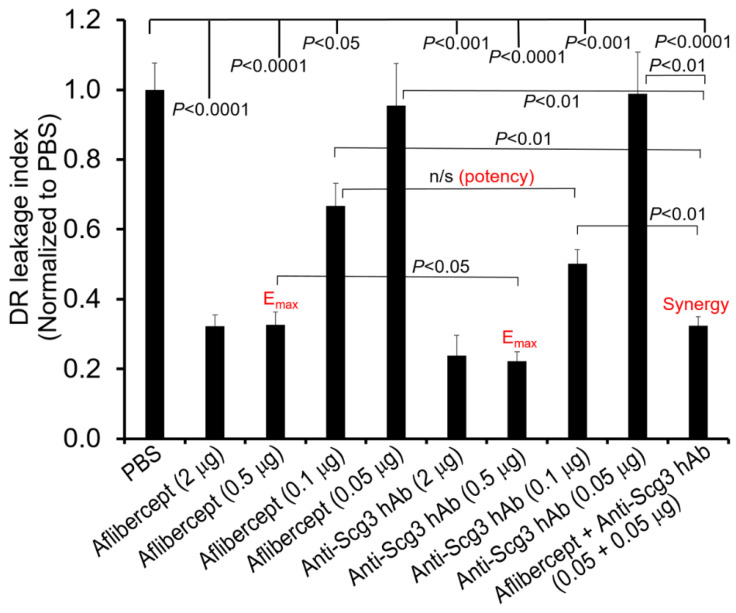
Synergistic combination therapy. Anti-Scg3 hAb or aflibercept was ivt injected into one eye at indicated doses with PBS for the fellow eye. After 24 h, DR leakage was quantified using Evans blue assay and normalized to fellow PBS eyes. + SEM. blind-coded. n = 5 mice/group, blind-coded. All data are normalized and compared against PBS fellow eyes of the same mice by paired *t*-test, as indicated by the lines on the top of the figure. Other comparisons indicated by individual brackets are analyzed using one-way ANOVA tests.

Our binding studies suggest that selective binding of Scg3 to the deep retinal plexus is predominantly responsible for DR-related vascular leakage, and therefore we asked whether such leakage was also neutralized by anti-Scg3 hAb. Dose–response curves comparing relative efficacies of anti-Scg3 hAb and aflibercept to ameliorate DR leakage are shown in Figure 5. We found that both Scg3 hAb and aflibercept dose-dependently inhibited such leakage with maximal efficacies (E_max_) of ~0.5 µg/eye and half maximal effective doses (EC_50_) of ~0.1 µg/eye. At E_max_, anti-Scg3 hAb was significantly more effective than aflibercept (*p* < 0.05), whereas at the EC_50_ dose, anti-Scg3 hAb was slightly more effective than aflibercept but did not meet statistical significance (*p* > 0.05). Therefore, anti-Scg3 hAb and aflibercept alleviate DR-related vascular leakage with similar potencies in this model.

### 2.7. Scg3 and VEGF Differentially Induce Calcium Influx

**Figure 6:**
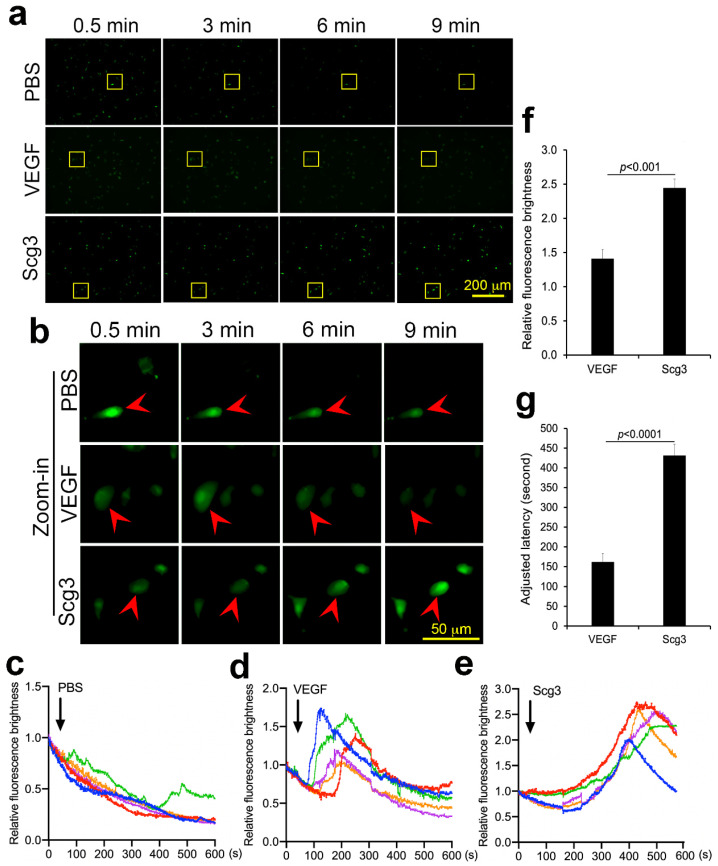
Differential calcium influx induced by Scg3 vs. VEGF in HRMEVCs. (**a**) Representative image of cells with calcium probe Fluo-8 AM at different times after treatment with Scg3 (1 µg/mL), VEGF (100 ng/mL) or PBS. (**b**) Zoom-in images of individual cells. Arrowheads indicate individual cells with the calcium probe. (**c**–**e**) Recording of the fluorescent intensity of the calcium probe in individual cells treated with PBS (**c**), VEGF (**d**) and Scg3 (**e**). Each line represents a single cell. (**f**) Quantification of the peak fluorescence intensity for VEGF and Scg3. (**g**) Quantification of latency for VEGF and Scg3. Yellow boxes in (**a**) are areas for cognate zoom-in images in (**b**). Colored lines in (**c**–**e**) represent individual cells.

Because calcium handling has been variously linked with the integrity of the retinal microcirculation and its disruption during DR [20], we compared basic calcium influx kinetics in HRMVECs driven by VEGF and Scg3. After stimulation with Scg3, VEGF or PBS, calcium influxes were tracked using a Fluo-8 AM calcium probe. Simultaneous tracking of five responsive cells in each group revealed that VEGF-induced calcium influx peaked between ~90 and 220 s after stimulation, whereas the Scg3-induced calcium influx was maximal between ~370 and 480 s and generated a more robust signal increase; both were significant (*p* < 0.001, Figure 6). The latency difference suggests that Scg3 and VEGF may regulate calcium influx through distinct signaling pathways.

### 2.8. Synergism of Anti-Scg3 and Anti-VEGF in Combination Therapy

Because of their different signaling pathways, we predicted and confirmed that the combination of anti-Scg3 hAb and aflibercept at subeffective doses (0.05 + 0.05 μg/1 μL/eye) conferred synergistic efficacy to ameliorate DR leakage (*p* < 0.01, vs. 0.5 μg/eye alone, Figure 5). The efficacy of aflibercept (0.05 µg/eye) was improved ~10x (i.e., equivalent to 0.5 µg/eye) by a subeffective dose of anti-Scg3 hAb in the combination therapy.

### 2.9. Reduced Severity of DR Leakage and Anti-Scg3 Therapy in Scg3^−/−^ Mice

**Figure 7:**
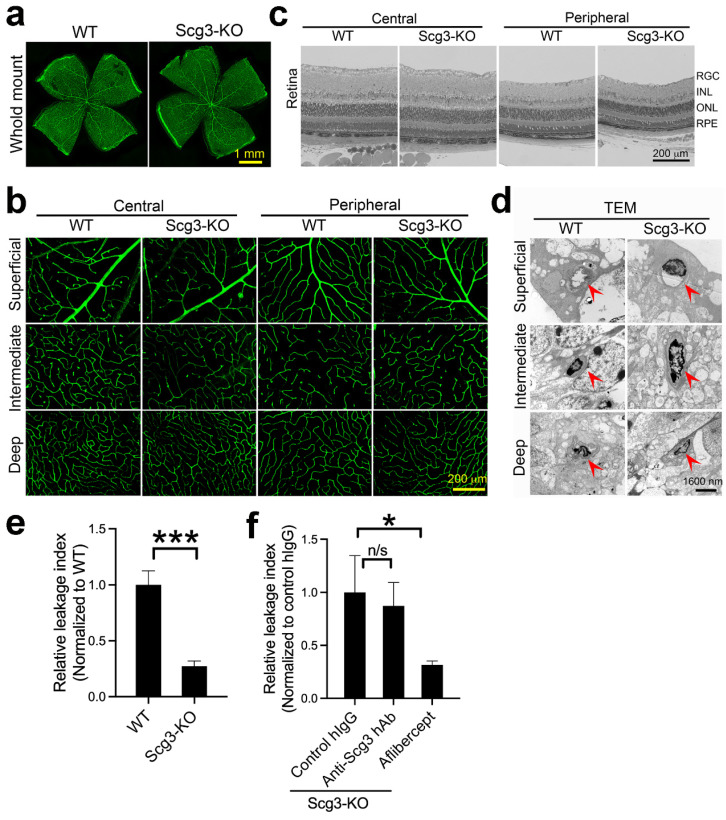
Severity of DR leakage and anti-Scg3 therapy in Scg3^−/−^ diabetic mice. (**a**) Comparison of the retinal vasculature in wild-type and Scg3^−/−^ mice. Flat-mounted retinas were stained with Alexa Fluor 488-isolectin B4 to visualize retinal vessels. (**b**) Comparison of the superficial, intermediate and deep retinal plexuses of wild-type and Scg3-deficient mice. Vessels of flat-mounted retinas were stained, as described in (**a**). (**c**) Comparison of retinal structures of wild-type and Scg3-knockout (KO) mice. (**d**) Representative images of retinal endothelial cells indicated by red arrows in the superficial, intermediate and deep retinal vascular plexuses of wild-type and Scg3-null mice by transmission electron microscopy. (**e**) Severity of DR leakage in wild-type and Scg3^−/−^ mice. Data were normalized to wild-type mice. n = 8 mice for wild-type and 6 mice for Scg3^−/−^ group. (**f**) Therapeutic efficacy of anti-Scg3 hAb and aflibercept (2 µg/1 µL/eye) in Scg3^−/−^ mice. Data were normalized to control hIgG (2 µg/1 µL/eye). n = 6 mice for control hIgG, n = 5 mice for anti-Scg3 hAb and n = 6 for aflibercept group. ± SEM. blind-coded. * *p* < 0.05, *** *p* < 0.001, n/s, not significant; one-way ANOVA test.

As a second approach to characterize the apparent selective retinal targeting of Scg3 and its predominant role in DR-related vascular leakage, we compared retinal vascular structures of wild-type and Scg3-deficient (Scg3^−/−^) mice. These mice were previously shown to present normal viability, body weight, behavior, fertility, and retinal development [18,21,22]. As shown in Figure 7a, histological analyses of vascular structures in flat-mount retinas revealed no differences between wild-type and Scg3-deficient mice. This conclusion was supported by high-magnification analyses of the superficial, intermediate and deep plexuses in the central and peripheral retina, which also revealed no differences (Figure 7b).

Retinas embedded in plastic resin showed similar structural integrity of the central and peripheral retina in Scg3^+/+^ and Scg3^−/−^ mice under low amplification (Figure 7c). Transmission electron microscopy revealed no differences between these two strains of mice in the endothelial structure of the superficial, intermediate and deep plexuses (Figure 7d).

Finally, in agreement with our proposed critical role for Scg3 in DR pathogenesis, Scg3^−/−^ mice presented significantly reduced severity of DR leakage (Figure 7e). As expected, aflibercept but not anti-Scg3 hAb significantly alleviated DR leakage in Scg3-null mice (Figure 7f). The results are consistent with our characterization of Scg3 as a disease-selective DR leakage factor that does not affect vascular function or integrity in healthy retinas.

## 3. Discussion

By conveying superior efficacy over available alternatives, anti-VEGF drugs are an available treatment for DR but have limitations, such as poor or absent responses in 15–40% of patients [8,9]. VEGF plays central roles in regulating both physiological and pathological angiogenesis [23], including vascular remodeling of adult retinas [24,25]. As with most drugs, the therapeutic value of anti-VEGF is a function of positive therapeutic benefits versus adverse side effects [26]. In animal models, systemic or ivt VEGF inhibitors reduce the density of healthy vasculatures [17,18,27,28]. Increased non-perfusion areas, foveal avascular zones and retinal ischemia as well as reduced retinal vascular density have been reported in some clinical studies of anti-VEGF therapy for DME and wet age-related macular degeneration (AMD) [29,30,31,32]. Such effects on healthy retinal vessels may contribute to the limited improvements in visual acuity of DME patients seen in clinical trials and practice of anti-VEGF therapy [8,9]. By sparing healthy vessels, selective targeting of diseased vasculatures is predicted to circumvent such adverse side effects. To our knowledge, all current anti-angiogenic/leakage therapies, which are approved or in the pipeline, indiscriminately inhibit both diseased and healthy vessels. We recently reported that Scg3 binds selectively to diseased but not healthy retinas, and that anti-Scg3 hAb selectively inhibits pathological but not physiological angiogenesis with no detectable side effects on healthy vessels in animal models of ROP [18]. In contrast, aflibercept indiscriminately inhibits angiogenesis and may confer adverse side effects [18]. This study reports a similar disease-restricted activity for Scg3, but not VEGF, to stimulate retinal vascular leakage in diabetic mice (Figure 2), which is consistent with our previous studies [16]. We further extend this work to identification of the deep retinal plexus as the exclusive binding site for Scg3 in DR mice and evidence that such binding sites are responsible for initiating and propagating pathological vascular leakage during DR.

The retinal deep plexus and choriocapillaris supply oxygen by diffusion to photoreceptors in the avascular outer half of the retina [6]. With high energy demands, photoreceptors are rich in mitochondria and major sources of both superoxide and pro-inflammatory products during DR that can destabilize proximal retinal blood vessels [33]. We propose that such photoreceptor-derived products selectively engage the vessels of the deep plexus and choriocapillaris, causing the loss of CD31 and activation of Scg3Rs with vascular leakage. This is consistent with reports of capillary density changes in the deep retinal plexus that correlate with DR severity and visual acuity in patients [34,35,36]. Previous work documented downregulation of endothelial CD31 in DR vasculatures that paralleled increased endothelial permeability [37]. In our studies, Scg3 bound only to CD31^−^ endothelium in the deep plexus of DR mice (Figure 3b and Figure 4), such that loss of endothelial integrity also correlates with Scg3 binding. The causal nature of such a correlation is supported by the actions of Scg3-neutralizing antibodies that block binding and preserve endothelial integrity (Figure 3b, Figure 4b, Figure 5 and Figure 7). A similar scenario seems possible for the choriocapillaris wherein Scg3 binding is also increased, albeit to a lesser degree (Figure 3b vs. Figure 3c) [38,39]. Breakdown of the blood-retina barrier (BRB) in the deep but not superficial plexus may explain why fluid accumulates in the intraretinal space with retinal thickening and DME rather than in the vitreous. While our results implicate overriding contributions of the deep plexus to vascular leakage in early DR mediated by Scg3, the intermediate and superficial plexuses are known to be involved at later stages.

In contrast to Scg3, VEGF drives DR-associated vascular pathology primarily through increased levels of ligand with estimates of a >100-fold increase in VEGF-A in the vitreous of PDR patients [40,41]. The low specificity and absence of apparent DR-related differential binding of VEGF observed in our study is consistent with other reports that VEGF receptors (VEGFRs) are minimally elevated or even decreased in diabetic rodents and patients [41,42]. The binding pattern is also consistent with the ubiquitous presence of VEGFRs in retinal vessels in control and DR mice, as discussed previously [15]. The differential binding profiles of Scg3 vs. VEGF (Figure 3 and Figure 4) reflect their distinctive mode of receptor signaling and highlight the unique disease-targeting property by anti-Scg3. Increased availability of Scg3Rs only on DR-stressed vessels with minimal induction of Scg3 ligand restricts Scg3 signaling to the vessels, thereby confining anti-Scg3 action to the deep plexus and by extension anti-Scg3 hAb therapy. In contrast, increased circulating VEGF activates ubiquitous VEGFRs on all retinal vessels, and VEGF blockers indiscriminately inhibit binding equivalently on DR-stressed and healthy retinal vasculatures. Stringent targeting of the deep plexus of DR retinas accounts for the minimal adverse effects of anti-Scg3 hAb on non-DR-stressed retinal vasculatures, thereby fulfilling the criteria for a disease-restricted therapy with the associated safety benefits. In this regard, we predict that disease-targeted anti-Scg3 does not increase non-perfusion areas, and foveal avascular zones by extension will provide optimal visual acuity improvements in DR.

Our functional studies showing synergy between anti-Scg3 and anti-VEGF in blocking DR leakage support the feasibility of a combination therapy. Previous attempts at such strategies have been unsuccessful, which is at least in part because the targets usually cross-react with VEGF signaling so that antagonists lack synergy with anti-VEGF. For example, faricimab that simultaneously targets VEGF and angiopoietin-2 was recently approved, not because it was superior to aflibercept alone but because Phase III trials of DME and wet AMD showed non-inferiority to anti-VEGF [43,44]. In contrast, our studies demonstrate a clear synergy between anti-Scg3 hAb and aflibercept (Figure 5). Such synergy is supported by differences in the latency and brightness of Scg3- and VEGF-induced calcium influx (Figure 6) that imply distinctive receptor signaling pathways for vascular leakage, as previously reported by our group [15]. This result is also consistent with the respective differential receptor binding activity patterns of VEGF and Scg3 (Figure 3b,d and Figure 4). Such a combination therapy is urgently needed for DR patients who respond poorly and/or are resistant to anti-VEGF monotherapy.

Identification of Scg3 binding to the deep plexus provides further validation of the novel binding assays developed by our group to screen for functional ligands in vivo [18,21,45]. Using the same techniques in a mouse model of ROP, we recently reported clustered binding of Scg3 to the superficial plexus coincident with pathological retinal neovascularization [18]. It is possible that clustered Scg3-Scg3R binding signals vessel sprouting in endothelium of the ROP superficial plexus, whereas more diffused Scg3 binding to the deep retinal plexus of DR only stimulates vascular leakage (Figure 2 and Figure 4).

In conclusion, we report that CD31^−^ endothelium of the deep retinal plexus selectively bind Scg3 in response to early DR stress, coinciding with the onset of compromised endothelial permeability. Because of its location, the deep retinal plexus may be especially vulnerable to diabetes-related metabolites that diffuse from DR-stressed photoreceptors. By selectively targeting the DR-stressed deep plexus, anti-Scg3 hAb represents a next-generation disease-targeted anti-angiogenic/leakage therapy, predicted to incur minimal side effects on relatively healthy plexuses in DR patients. By virtue of distinct pro-angiogenic pathways and synergistic therapeutic actions, combination of anti-Scg3 and anti-VEGF may improve treatment of patients who respond poorly to sole anti-VEGF therapy. The results support clinical development of such a strategy.

## 4. Materials and Methods

### 4.1. Animals and Materials

C57BL/6J mice were purchased from the Jackson Laboratory. Scg3^−/−^ mice were previously described [21]. Mice (6–8 weeks old, male) were treated with streptozotocin to induce hyperglycemia (blood glucose > 300 mg/dL) and aged for 4 months to develop chronic DR [15]. All animals were randomly assigned to each group. All therapeutic reagents were blind-coded, as indicated. All procedures of animal studies were approved by the Institutional Animal Care and Use Committee at Baylor College of Medicine.

Scg3-neutralizing hAb and related Fab fragment (hFab) were generated from an optimal Scg3-neutralizing mAb by Everglades Biopharma, LLC. Human retinal microvascular endothelial cells (HRMVECs) were obtained from Cell Systems [15].

### 4.2. In Vivo Ligand Binding Assay

This assay was performed as described [18,21,45]. Briefly, clonal T7 phages displaying VEGF_110_ (VEGF-Phage) and full-length human Scg3 (Scg3-Phage) were amplified, purified, dialyzed against PBS, titrated by plaque assay, and preincubated with or without anti-Scg3 hFab or aflibercept (Regeneron Pharmaceuticals) (4 µg/mL, each), respectively, at 4 °C for 30 min. Purified phages were injected intravenously (i.v.) into anesthetized diabetic or healthy mice (1 × 10^12^ plaque forming unit (pfu)/200 μL/mouse). After circulating for 20 min, mice were euthanized by CO_2_ inhalation, immediately followed by intracardial perfusion with 70 mL of PBS for 7 min to remove unbound phages. Retinas were isolated, weighed, and homogenized in PBS with 1% Triton X-100 using a polytron homogenizer until no solid tissues were visible. Vessel-bound phages in homogenates were quantified by plaque assay.

### 4.3. Functional Immunohistochemistry (FIHC)

To improve detection sensitivity, the Scg3-Phage or VEGF-Phage was amplified in BLT7FLAG bacteria to label each phage particle with ~400 copies of FLAG tag [46]. Amplified phages were purified for in vivo ligand binding as above, followed by sequential intracardial perfusions with 40 mL PBS for 4 min, 20 mL of 4% PFA for 2 min and then 10 mL PBS for 1 min. Retinas were isolated, permeabilized, blocked in Solution A (5% goat serum and 1% Triton X-100 in PBS) overnight at 4 °C, and incubated with anti-FLAG M2 mAb (Sigma, St. Louis, MO, USA; #F1804; 1:200) and rabbit anti-CD31 pAb (Abcam, #28364; 1:50) in Solution A for 2 days at 4 °C. After washing, retinas were incubated with Alexa Fluor 594-conjugated anti-mouse IgG H&L (Cell Signaling, Danvers, MA, USA; #8890S; 1:1000) and Alexa Fluor 488-conjugated ani-rabbit IgG (H + L) (Cell Signaling, #4412S, 1:1000) in Solution A overnight at 4 °C. Retinas were washed, flat-mounted in 50% glycerol in PBS and viewed under a Keyence structured illumination fluorescence microscope (SIM; Osaka, Japan; Model BZ-X800).

### 4.4. Calcium Influx Assay

HRMVECs (Cell Systems, Kirkland, WA, USA) were harvested and incubated with Fluo-8 AM (5 µg/mL; Abcam, Cambridge, UK; #ab142773) at 37 °C for 1 h, followed by washing with Hanks’ balanced salt solution (HBSS) for 3 times × 3 min. HRMVECs in PBS were recorded for 10 min using the SIM. The VEGF (R&D Systems, Minneapolis, MN, USA; #BT-VEGF-100), Scg3 (Sino Biological, Wayne, PA, USA; #16012-H08H) or PBS was added at 30 s after starting the recording, as described [47].

### 4.5. In Vivo FITC-dextran Leakage Assay

The VEGF (100 ng) or Scg3 (250 ng) was injected intravitreally (ivt) into diabetic or healthy mice. After 24 h, FITC-dextran (70 kDa, Sigma, Cat #46945) was injected i.v. into mice (500 mg/mL, 200 µL/20 g body weight). After circulation for 2 min, mice were euthanized by CO_2_ inhalation. Eyes were enucleated and immediately embedded in an optimal cutting temperature compound. Cryosections of 10-µm thickness were immediately viewed and photographed under the SIM, as described [48]. FITC leakage spots per viewing field were quantified. FITC leakage intensity was quantified using ImageJ (NIH).

### 4.6. DR Therapy

Anti-Scg3 hAb, human control IgG (Sigma), aflibercept (1 µL/eye) or PBS was blind-coded and injected ivt into diabetic mice with PBS always for the fellow eye. After 16 h, retinal vascular leakage was quantified by Evans blue assay and normalized against the blood level of Evans blue, retinal weight and PBS in the fellow eye, as previously described [15]. We noticed that occasional outlier mice with a dramatic increase in DR leakage of the PBS control eye may be detected, albeit at an extremely low frequency. The exclusion criterion of outliers was any DR mice whose normalized Evans blue leakage index in PBS fellow eyes is >± 5X SD of all DR PBS eyes.

### 4.7. Statistics

Data were expressed as mean ± SEM. Intergroup differences were analyzed through one-way ANOVAs or Student’s *t*-tests.

## Data Availability

All data generated or analyzed in this study are included in this article and its Supplementary Materials.

## Supplementary Materials

The following supporting information can be downloaded at: https://www.mdpi.com/article/10.3390/ijms241310531/s1. References [1,15,50] are cited in the supplementary materials.
