# Supplementary material for: Secretogranin III Selectively Promotes Vascular Leakage in the Deep Vascular Plexus of Diabetic Retinopathy
Source: Int J Mol Sci. 2023 Jun 23;24(13):10531. doi: 10.3390/ijms241310531 (PMC10341987; doi:10.3390/ijms241310531)
Supplement: Supplementary file 1 [file ijms-24-10531-s001.zip › ijms-2370250-supplementary.pdf]

Supplementary Material for

Scg3 selectively promotes vascular leakage in the deep vascular plexus of diabetic retinopathy

Liyang Ji, Prabuddha Waduge, Yan Wu, Chengchi Huang, Avinash Kaur, Paola Oliveira, Hong Tian, Jinsong Zhang, J. Timothy Stout, Christina Y. Weng, Keith A. Webster, Wei Li

Corresponding author: wei.li4@bcm.edu (W.L.)

The PDF file includes

Supplementary Methods

References for Supplementary Methods

Supplementary Figures S1 - S8

Supplementary Methods

Immunohistochemistry

Diabetic mice were euthanized by CO_2 inhalation and intracardially perfused with 20 ml PBS for 2 min, followed by 10 ml 4% paraformaldehyde (PFA) for 1 min. Enucleated eyes without the anterior section were refixed in 4% PFA for 40 min at room temperature, dehydrated to 30% sucrose [1], embedded in optimal cutting temperature compound. Human retinas with or without diabetes for more than 10 years were acquired from Lions Eye Bank of Texas, fixed and embedded in a similar manner. Cryosections of eyes in 6-µm thickness were co-stained with anti-Scg3 rabbit polyclonal antibody (Proteintech, #10954-1-AP; 1:100) [15] and anti-CD31 mouse mAb (Abcam, #ab24590; 1:200) in Solution A (5% goat serum and 0.3% Triton X-100 in PBS). After washing, bound antibodies were visualized using Alexa Fluor 594-conjugated goat anti-rabbit IgG antibody (Cell Signaling, #8899S, 1:1,000) and Alexa Fluor 488-conjugated goat anti-mouse IgG (H+L) antibody (Cell Signaling, #4408S, 1:1,000) in Solution A. Nuclei were stained with DAPI. Signals were analyzed using a Keyence structured illumination fluorescence microscope (SIM, Model BZ-X800).

Additionally, retinas were isolated from wild-type or Scg3$^{-/-}$ mice, fixed in 4% PFA for 40 min, permeabilized in PBS with 0.5% Triton X-100 overnight at 4°C and incubated with Alexa Fluor 488-isolectin B4 (10 µg/ml, Thermo Fisher Scientific, #I21411) overnight in PBS with 1 mM $CaCl_2$ and 2 mM NaN_3 at room temperature. After refixation in 4% PFA, flat-mount retinas were analyzed using the SIM microscope.

Transwell endothelial cell migration

Transwell migration assay was performed, as described [50]. Briefly, Scg3 (1 µg/ml) or VEGF (100 ng/ml) was incubated with HRMVECs (3 x 10^4 cells/well, free of mycoplasma) in the transwell inserts of 24-well plates (8 µm pore size, Corning cat #3422) in the presence or absence of anti-Scg3 hFab (3 µg/ml) or aflibercept (1 µg/ml), respectively. HRMVECs on the upper side of the membrane were removed using Q-tips at 20 h. Cells migrated to the bottom side of the membrane were stained with DAPI and quantified under the SIM.

Transwell permeability assay

Transwell permeability assay was performed, as described [15]. HRMVECs (5 x 10^4 cells/well) were seeded on transwell inserts of 24-well plates (0.4 µm pore size, Corning Life Science, #3470) precoated with 1% gelatin. After overnight culture to form transwell monolayer, VEGF (100 ng/ml) or Scg3 (1 µg/ml) was preincubated with aflibercept (1.5 µg/ml) or anti-Scg3 hAb (5 µg/ml), respectively, for 30 min and added to the lower chamber along with FITC-dextran (0.5 mg/ml, 70 kDa, Sigma, #46945). PBS was included as a control. Media in the upper chamber were collected at 4 h, quantified for FITC and compared against a standard curve to calculate leaked FITC-dextran.

Tube formation assay

The assay was described previously [15]. Briefly, 96-well plates were coated with Matrigel (100 µl/well, Corning Life Science, #354263). HRMVECs were starved in serum-free EBM-2 medium (Lonza) for 3 h, harvested and plated on Matrigel-coated 96-well plates (15,000 cells/well). Cells were incubated with Scg3 (300 ng/ml), VEGF (50 ng/ml) or PBS in EBM-2 medium at 37°C in the presence or absence of anti-Scg3 hAb (1.5 µg/ml) or aflibercept (500 ng/ml), as indicated. After incubation for 4 h, bright field images were taken. Total tube length, number of tubes and number of branching points per viewing field were quantified using ImageJ software (NIH).

Transendothelial electrical resistance (TEER) assay

HRMVECs (5 x 10^4 cells/well) were seeded on transwell inserts of 24-well plates (Corning Life Science, #3470) precoated with 1% gelatin. After overnight culture, transmonolayer specific resistance (Ω•cm^2) was measured using EVOM2 Epithelial Volt/Ohm Meter (World Precision Instrument) and compared to cell-free control wells to confirm the formation of the monolayer [51]. VEGF (100 ng/ml) or Scg3 (1 µg/ml) was preincubated with or without aflibercept (1.5 µg/ml) or anti-Scg3 hFab (5 µg/ml), respectively, for 30 min and added to the upper chamber. PBS was included as a control. Transwell resistance was measured 4 h afterward.

Electron microscopy

Eyes were enucleated from euthanized healthy $Scg3^{-/-}$ and $Scg3^{+/+}$ mice at 7 weeks old. After the removal of the anterior section, eyes were fixed in 3% glutaraldehyde, washed in 0.1 M

sodium phosphate buffer (pH 7.3), re-fixed in 1% osmium tetroxide and dehydrated in a series of graded ethanol solutions to a final 100% ethanol. Eyes were infiltrated (harden) with acetone and embedded in Poly/Bed® 812 plastic resin. Sections (80-90 nm) cut on a Leica Ultracut R ultramicrotome were stained with uranyl acetate and lead citrate and analyzed using a Zeiss EM902 Transmission Electron Microscope. Images were captured using an AMT V602 digital camera.

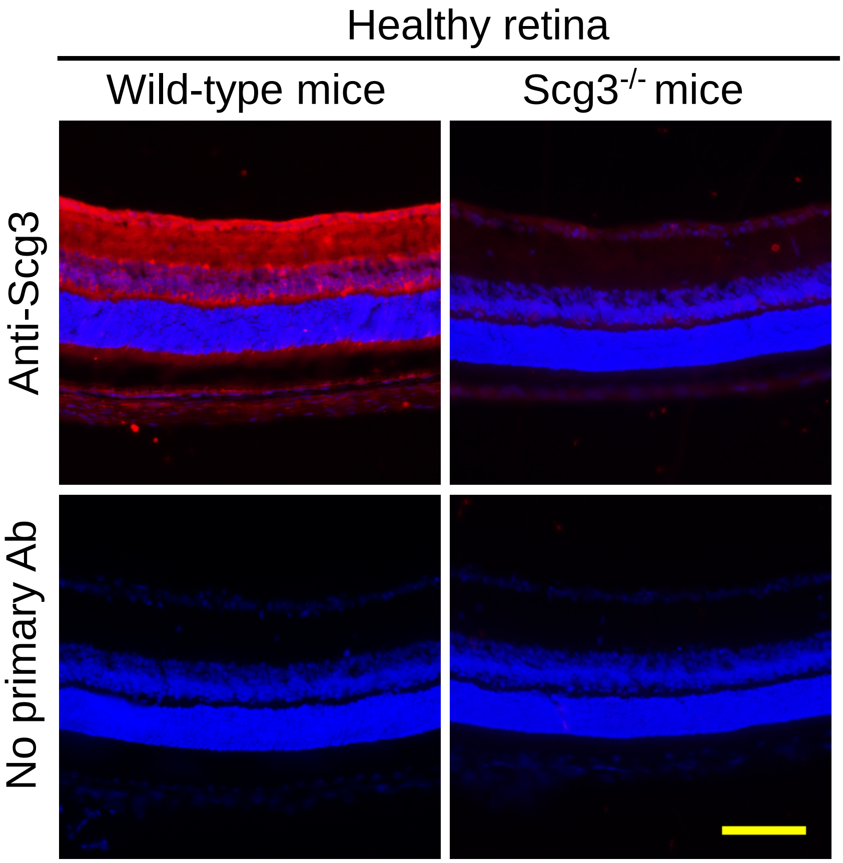

Supplementary Figure S1. Scg3 is expressed only in the retina of wild-type but not Scg3$^{-/-}$ mice. Scg3 was detected, as described in Figure 1a. Nuclei were stained with Hoechst. Scale bar = 100 μm.

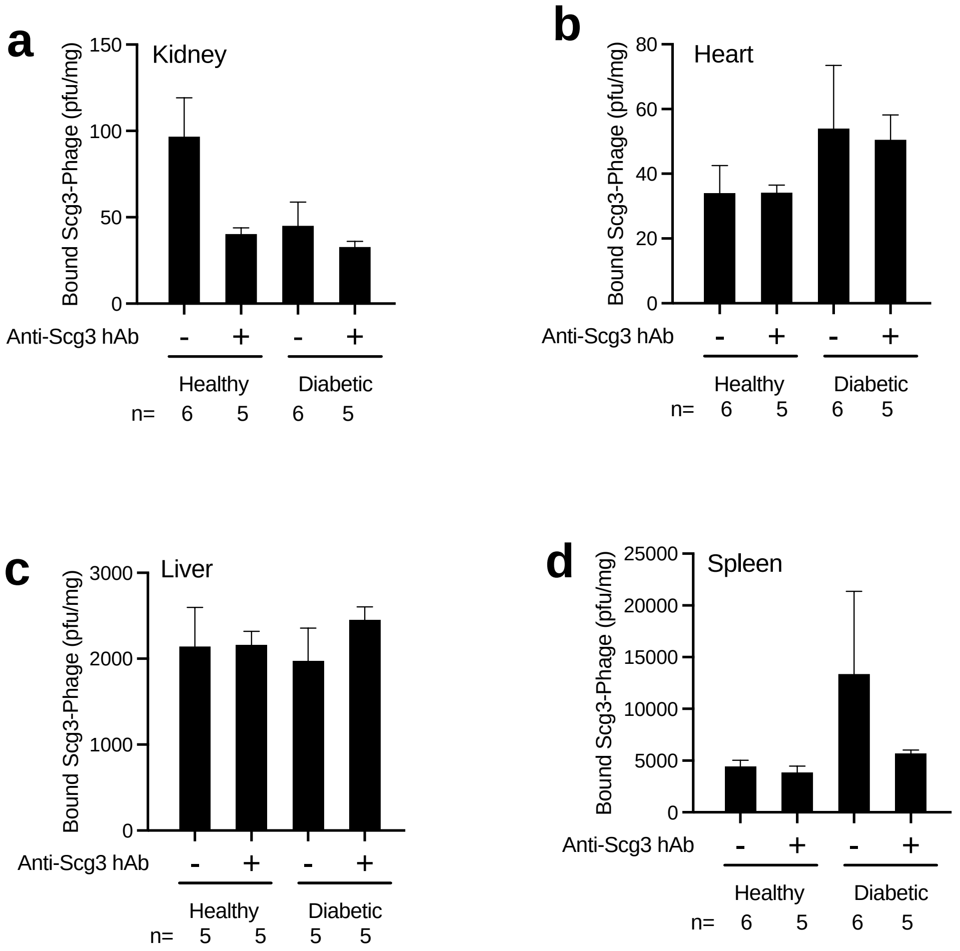

Supplementary Figure S2. Scg3 binding to non-ocular vasculatures in different organs of diabetic and healthy mice. Scg3 binding to the kidney **(a)**, heart **(b)**, live **(c)** and spleen **(d)** was quantified as in Figure 3b-e. Sample size (mice/group) is indicated at the bottom of the graph. ±SEM; t-test.

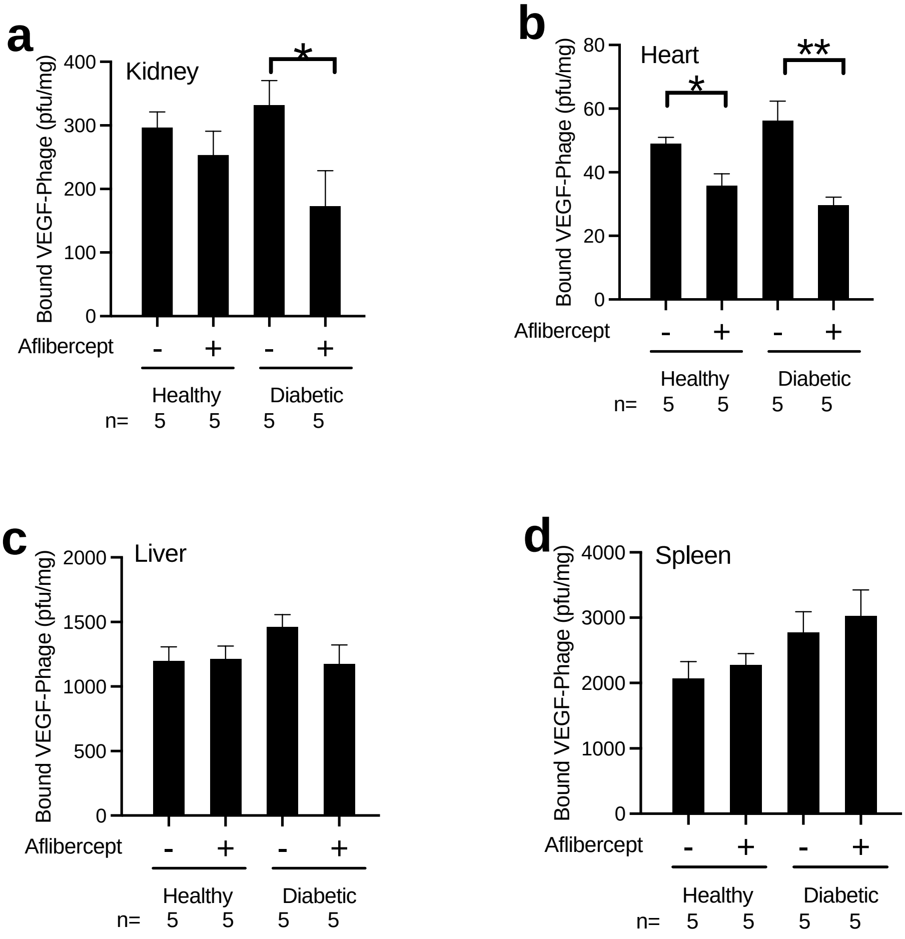

Supplementary Figure S3. VEGF binding to non-ocular vasculatures in different organs of diabetic and healthy mice. VEGF binding to the kidney **(a)**, heart **(b)**, live **(c)** and spleen **(d)** was quantified as in Figure 3b-e. n = 5 mice/group; ±SEM; * p<0.05, ** p<0.01; t-test.

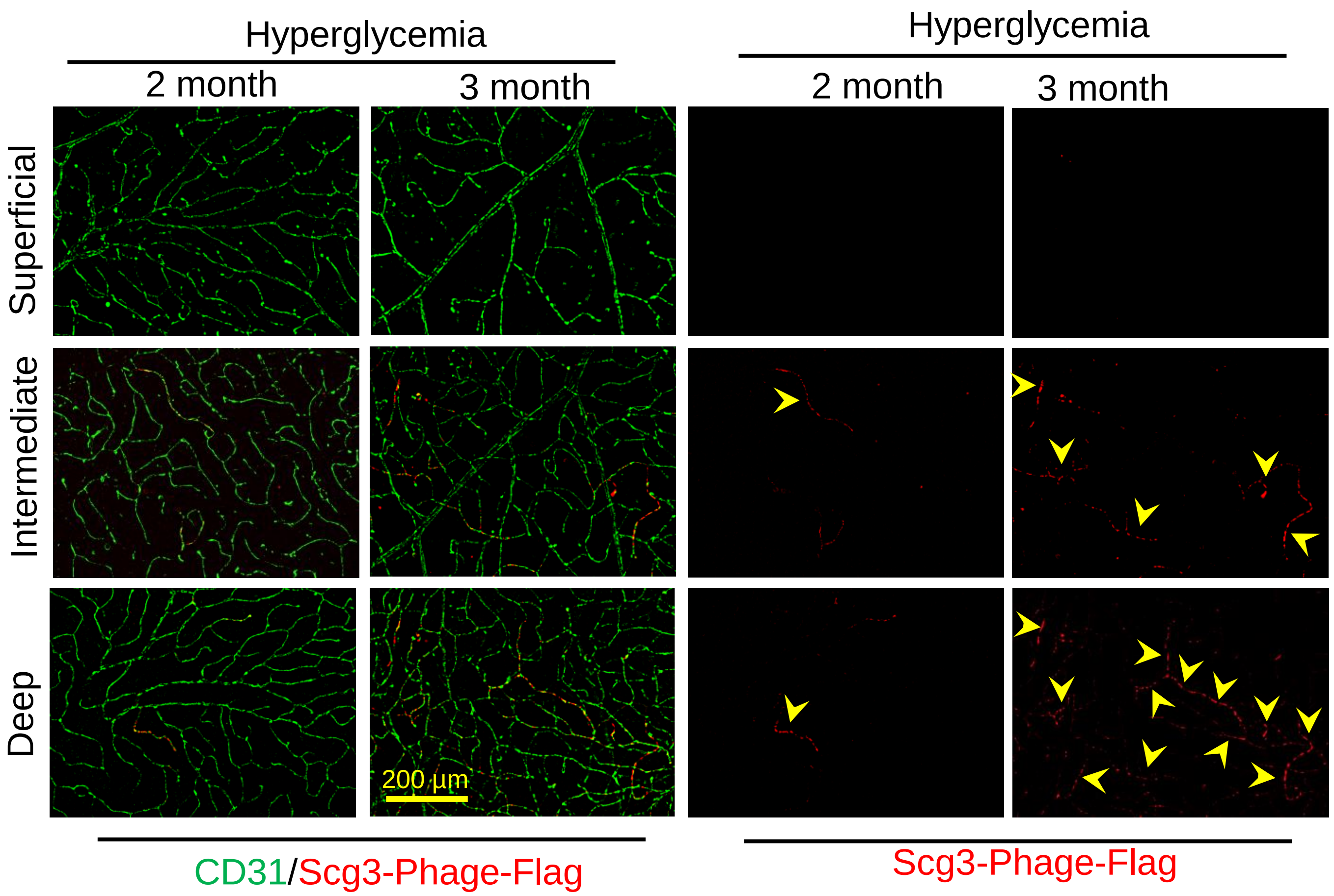

Supplementary Figure S4. Scg3 binding to 2- and 3-month-diabetic retina by FIHC. Scg3 binding to the retina of 2- and 3-month hyperglycemic mice was detected as described in Figure 2. Arrowheads indicate Scg3 binding signals.

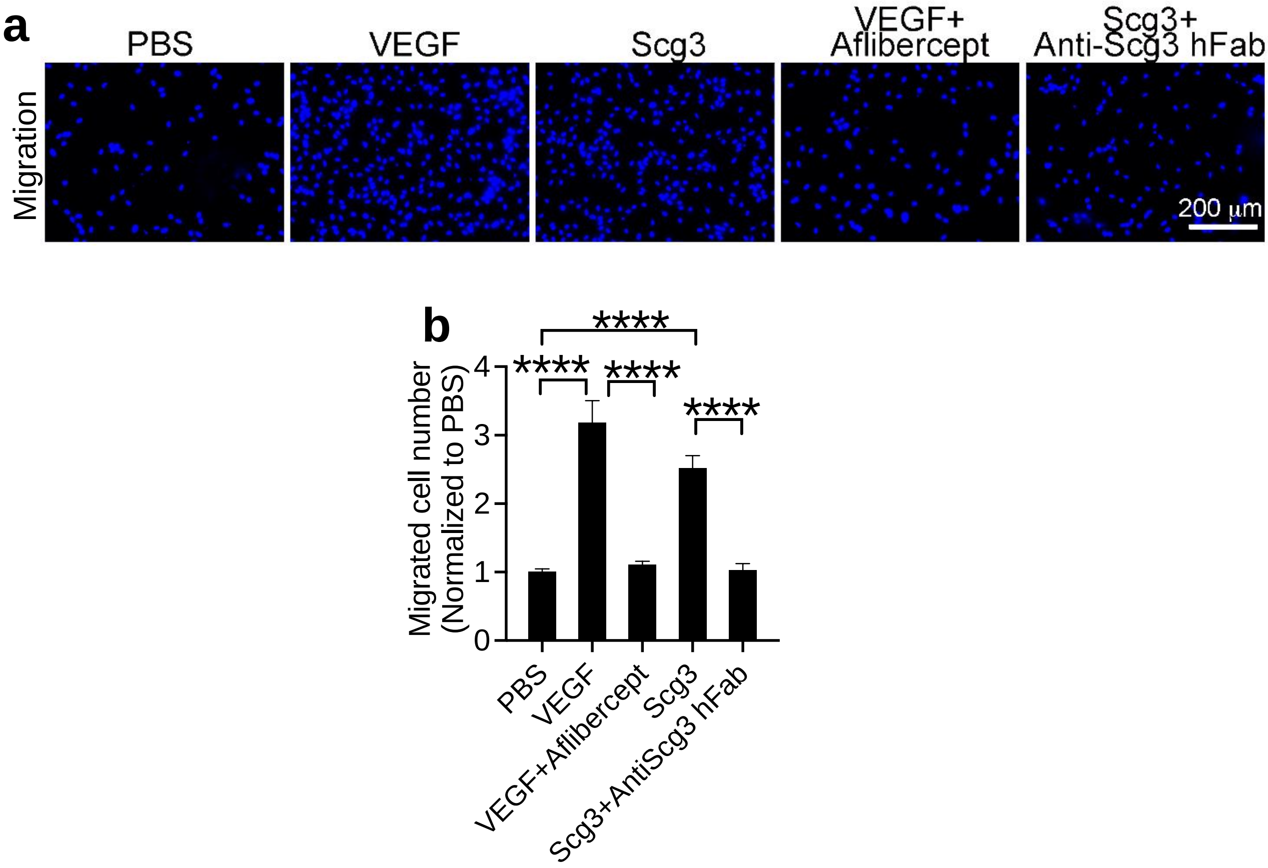

Supplementary Figure S5. *In vitro* trans-well endothelial migration assay to quantify neutralizing activity of anti-Scg3 hAb. **(a)** Anti-Scg3 hFab blocks Scg3-induced migration of HRMVECs. HRMVECs seeded on transwell membranes were stimulated with VEGF or Scg3 in the presence or absence of their cognate inhibitor, aflibercept or anti-Scg3 hFab, respectively. After 20 h, cells migrated to the bottom side of the membrane were stained with DAPI and analyzed. **(b)** Quantification of migrated cells in **(a)**. n=6 wells/group. ±SEM; **** $p<0.0001$; one-way ANOVA test.

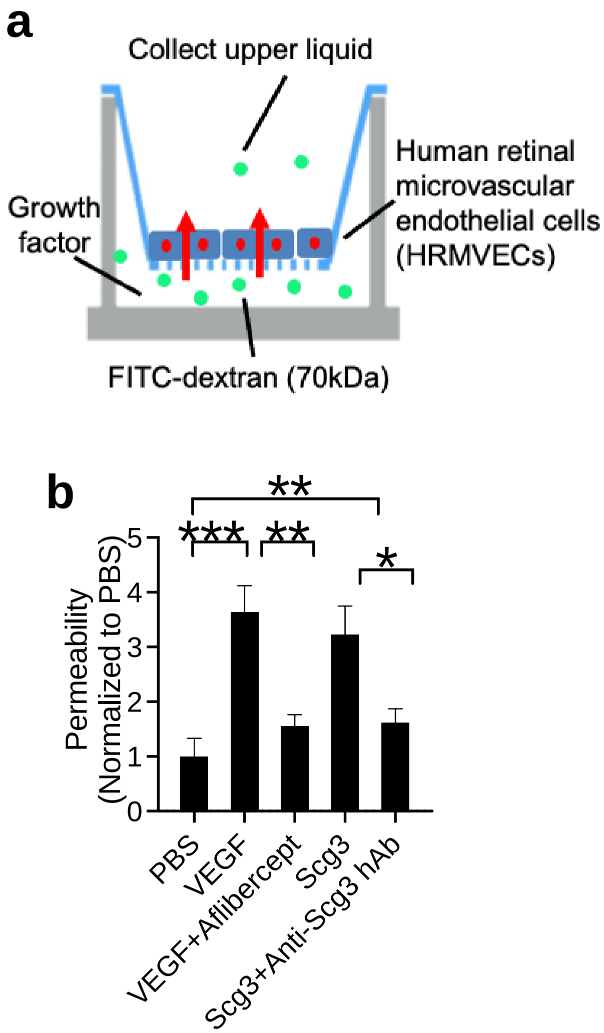

Supplementary Figure S6. *In vitro* trans-well endothelial permeability assay to quantify neutralizing activity of anti-Scg3 hAb. **(a)** Schematic of transwell permeability assay. HRMVECs were seeded on transwell membranes to form a monolayer. FITC-dextran (70 kDa), growth factors and their cognate blockers, including VEGF, Scg3, aflibercept and anti-Scg3 hFab, were added to the lower chamber. **(b)** Quantification of FITC-dextran in **(a)** after 4 h. n=5 wells/group. ±SEM; * $p<0.05$, ** $p<0.01$, *** $p<0.001$; one-way ANOVA test.

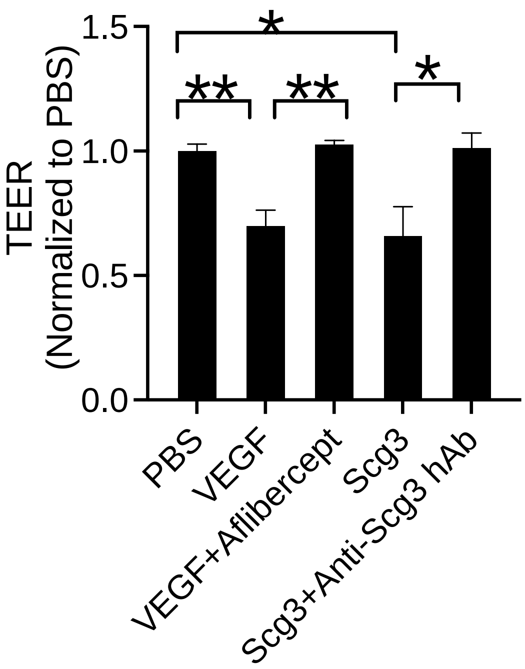

Supplementary Figure S7. Transendothelial electrical resistance (TEER) assay to quantify neutralizing activity of anti-Scg3 hAb. The assay weas performed in the presence or absence of VEGF, Scg3, aflibercept and anti-Scg3 hFab, as indicated. n=5 wells/group. ±SEM; * $p<0.05$, ** $p<0.01$; one-way ANOVA test.

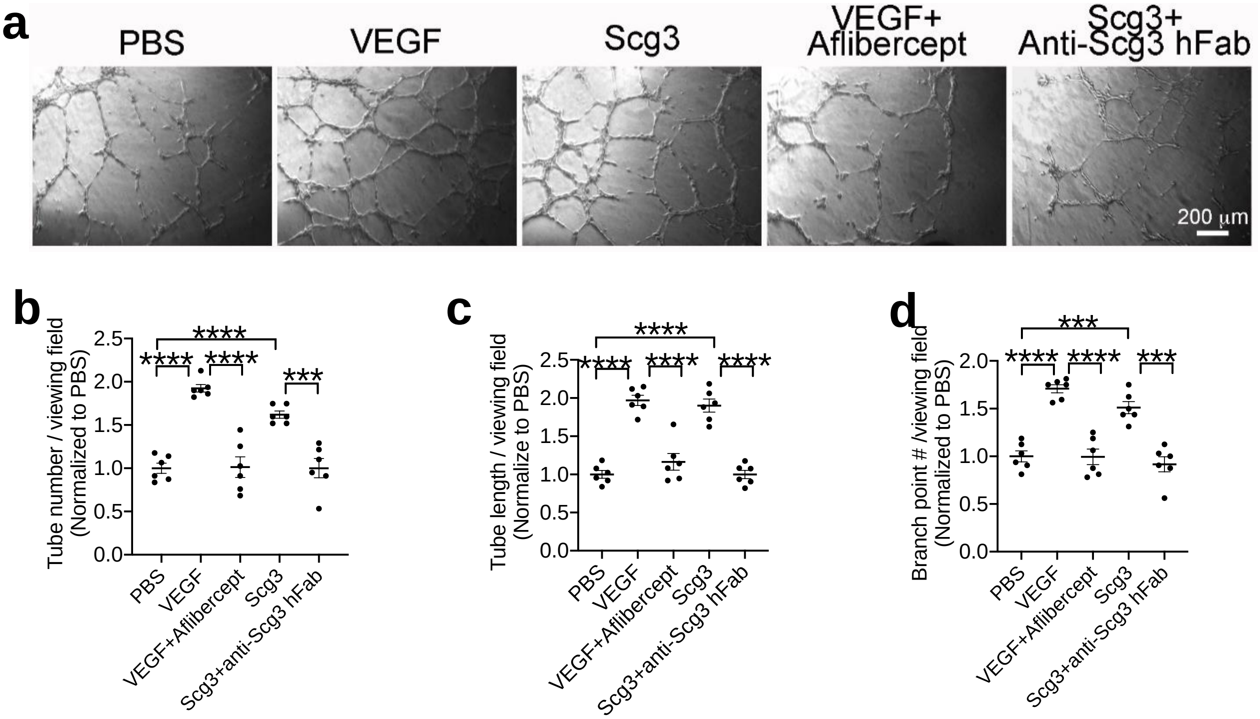

Supplementary Figure S8. *In vitro* Tube formation assay to quantify neutralizing activity of anti-Scg3 hAb. **(a)** The assay was performed using HRMVECs in the presence or absence of VEGF, Scg3, aflibercept and anti-Scg3 hFab, as indicated. **(b)** Quantification of tube number in (a). **(c)** Quantification of tube length in (a). **(d)** Quantification of branch points in (a). n=6 wells/group. ±SEM; *** $p<0.001$, **** $p<0.0001$; one-way ANOVA test.
